# Response of Poplar and Associated Fungal Endophytic Communities to a PAH Contamination Gradient

**DOI:** 10.3390/ijms23115909

**Published:** 2022-05-25

**Authors:** Lilian Gréau, Damien Blaudez, Dimitri Heintz, Julie Zumsteg, David Billet, Aurélie Cébron

**Affiliations:** 1 Université de Lorraine, CNRS, LIEC, 54000 Nancy, France; lilian.greau@univ-lorraine.fr (L.G.); damien.blaudez@univ-lorraine.fr (D.B.); david.billet@univ-lorraine.fr (D.B.); 2 Institut de Biologie Moléculaire des Plantes, CNRS, Université de Strasbourg, 67000 Strasbourg, France; dimitri.heintz@ibmp-cnrs.unistra.fr (D.H.); julie.zumsteg@ibmp-cnrs.unistra.fr (J.Z.); 3 Pôle de Compétences en Biologie Environnementale, Université de Lorraine, CNRS, LIEC, 54000 Nancy, France

**Keywords:** fungal communities, ITS sequencing, phenanthrene, phytotoxicity, soil pollution

## Abstract

Microbial populations associated to poplar are well described in non-contaminated and metal-contaminated environments but more poorly in the context of polycyclic aromatic hydrocarbon (PAH) contamination. This study aimed to understand how a gradient of phenanthrene (PHE) contamination affects poplar growth and the fungal microbiome in both soil and plant endosphere (roots, stems and leaves). Plant growth and fitness parameters indicated that the growth of *Populus canadensis* was impaired when PHE concentration increased above 400 mg kg^−1^. Values of alpha-diversity indicators of fungal diversity and richness were not affected by the PHE gradient. The PHE contamination had a stronger impact on the fungal community composition in the soil and root compartments compared to that of the aboveground organs. Most of the indicator species whose relative abundance was correlated with PHE contamination decreased along the gradient indicating a toxic effect of PHE on these fungal OTUs (Operational Taxonomic Units). However, the relative abundance of some OTUs such as *Cadophora*, *Alternaria* and *Aspergillus*, potentially linked to PHE degradation or being plant-beneficial taxa, increased along the gradient. Finally, this study allowed a deeper understanding of the dual response of plant and fungal communities in the case of a soil PAH contamination gradient leading to new perspectives on fungal assisted phytoremediation.

## 1. Introduction

Polycyclic aromatic hydrocarbons (PAHs) are ubiquitous environmental pollutants produced during incomplete combustion of organic material and generated from numerous anthropogenic industrial sources. Many PAHs have toxic, mutagenic and/or carcinogenic properties, and 16 PAH compounds are listed as priority pollutants by the United States Environmental Protection Agency (US-EPA) due to their high persistence and toxicity in the environment [1]. Although their dissipation is relatively slow in soils, the microbial biodegradation of PAHs is the main process leading to the mitigation of these organic pollutants [2,3].

The combined use of plants and their associated microbes is an appealing method for the ecological remediation of PAH-contaminated sites. This process called phytoremediation, which is assisted by microorganisms, makes use of the naturally occurring processes by which plants and their associated microbiome degrade pollutants [4]. Some studies have shown that PAH could be degraded more efficiently in planted than in unplanted soils [5] due to the rhizosphere effect [6]. For decades, PAHs were not expected to be susceptible to plant uptake and translocation because of their high hydrophobicity and adsorption to soil organic matter [7]. However, some studies have recently shown that PAHs could be taken up and translocated in plant tissues through a mechanism driven by transpiration from the belowground to the aboveground parts [8,9].

Because of their toxicity, we could hypothesize that PAH contaminants will induce a selection of microorganisms present in the soil and of the ones colonizing plant tissues, leading to a modification of the microbiome diversity. Only few studies have assessed plant-associated microbial taxonomic diversity and community shift in hydrocarbon contaminated sites [10]. Most of the studies on endophytes and their role in association with plants focused on bacterial endophytes, but fungal communities were also significantly impacted by hydrocarbon contamination in soils [11] and in plants [12].

Endophytic microorganisms, which are defined by any microbial genetic material found inside plant tissues [13], are also of interest in the context of phytoremediation, as successful phytoremediation systems are highly dependent on the plant-microbiome association fitness. Endophytes can colonize any plant tissue from the root tips to the leaves. Endophytes maintain a close relation with their host, frequently leading to tight beneficial relationships. Endophytes thrive from a protected and nutrient-rich environment conferred by the plant. In return, they produce a wide range of molecules improving plant growth and development by facilitating nutrient accessibility, producing phytohormones or exerting biocontrol activity [14,15,16,17,18]. Most studies focused on bacterial endophytes mitigating the role that fungi could play in these complex relationships between plants and their microbiota, especially in contaminated environments. Fungal endophytes can also metabolize PAHs [19]. Some studies reported that plant co-culture with fungal endophytes could increase PAH dissipation but also plant growth [20,21], indicating that the toxicity of PAHs to the plant can be mitigated by the microorganisms. Indeed, plants subjected to PAH pollution had their physiology affected [22], and the presence of some microorganisms will limit adverse effects. For instance, Sun and co-workers showed that ryegrass inoculated with bacterial strains grew better in a PAH contaminated soil compared to non-inoculated plants [23], but we need data on the beneficial effects of fungal taxa.

Poplar has been extensively studied for its phytoremediation capability due to an important root surface and high tolerance to a wide range of pollutants [24]. Poplar endophytome is well-described in non-anthropized soils [25,26] as well as in metal-contaminated ones [27,28]. However, the impact of organic pollutants and especially PAH contamination on the fungal soil microbiome and plant endophytome has been poorly studied. It is difficult to predict the effect of PAHs on the plant-microbiome association, because the toxic response most likely depends on the pollutant concentration, availability, and translocation in the plant tissues. Therefore, studying the effect of PAH contamination gradients could help highlighting this dose-response effect.

With the aim to study the impact of PAHs on plant growth and physiology and on associated fungal communities, an experimental design with a PAH contamination gradient was set up. We hypothesized that along a PAH gradient, the impact of the pollutant on the plant growth and fitness would be correlated to the level of the contaminant, and that PAH contamination would shape the soil microbiome by selecting most tolerant microbes at the expense of sensitive ones. Concerning the endophytic microbiome diversity, it would be shaped both by the plant fitness and by the level of pollutant translocated through the plant tissue. The main objective of our study was therefore to understand how an increasing range of PAH contamination (using phenanthrene, PHE, as a model PAH) affects poplar growth parameters and the diversity of the fungal microbiome in soil and the plant endosphere (roots, stems, leaves).

## 2. Results

### 2.1. Microscopic Evidence of Fungal Colonization

In the first microcosm experiment, the fungal colonization of *P. canadensis* roots was explored in both young and aged roots subjected to a PHE gradient (0; 200; 700; 1000; 1500 mg PHE kg^−1^; Figure S1). The fungal colonization rate in older roots was c.a. 20% whatever the PHE concentration. However, in young roots the fungal colonization was significantly lower and ranged from c.a. 8% to 0% (no fungal structure was observed) between low spiked PHE conditions (0 to 200 mg PHE kg^−1^) and highly spiked ones (700–1500 mg PHE kg^−1^), respectively (Figure S1). Different types of fungal structures were observed along the PHE gradient. At low PHE concentrations (0–200 mg PHE kg^−1^), we observed a high diversity of fungal structures such as typical hyphae, vesicles, arbuscular and *Olpidium*-like structures (Figure S1b–d). At high PHE concentrations (700–1000 mg PHE kg^−1^), a less important diversity of fungal structures was observed with mainly typical hyphae and sclerotia-like structures (Figure S1e). Based on this first experiment showing apparently different fungal diversity along the PHE gradient, we performed a second experiment with eight different concentrations of PHE to analyze the fungal diversity in soil and plant tissues and to evaluate the impact on plant growth parameters.

### 2.2. Impact of Phenanthrene Gradient on Plant Biomass and Fitness

**Figure 1:**
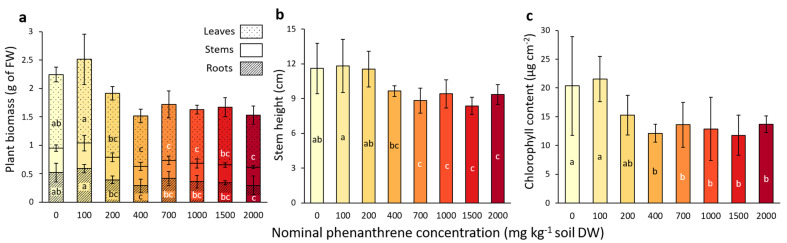
Plant growth parameters measured from poplar after four weeks of growth in a soil spiked with increasing phenanthrene concentrations. (**a**) Total biomass of leaf, stem and root compartments. (**b**) Stem height measurement. (**c**) Chlorophyll content in leaves measured with Dualex FORCE-A. The data represent the means of three to four plant replicates per condition and error bars represent standard deviation. When present, different letters indicate significant differences (*p* < 0.05) between conditions after one-way ANOVA (Analysis of variance) and Tukey’s *post-hoc* analysis, except for the leaf biomass, which was analysed by Kruskal-Wallis and Dunn *post-hoc* tests. For other parameters (leaf number, flavonols, anthocyanins and Nitrogen Balance Index), please refer to Table S1.

After four weeks of growth, the impact of the PHE toxicity on poplar fitness and growth was assessed. A significant decrease in leaf biomass was observed for concentrations above 400 mg kg^−1^ compared to the low-spiked PHE conditions (0–100 mg PHE kg^−1^ soil; Figure 1a). Concerning the root biomass, a significant decrease was observed for plants growing at 2000 mg PHE kg^−1^ soil compared with low-spiked PHE conditions (0–100 mg PHE kg^−1^ soil). The stem biomass was not significantly impacted by the PHE gradient, whereas stem height was significantly lower for concentrations above 700 mg kg^−1^ (Figure 1a,b) compared to lower-spiked PHE conditions (0–200 mg PHE kg^−1^ soil).

The chlorophyll content of the leaves (Figure 1c) significantly decreased for the plants that grew in a PHE spiked-soil with a concentration above 400 mg PHE kg^−1^ soil, compared to the lower-spiked conditions (0–100 mg PHE kg^−1^ soil). No significant differences were detected for anthocyan and flavanol contents in the leaves (Table S1), while the NBI (nitrogen balance index), an indicator of the plant nitrogen status combining chlorophylls and flavonols (related to nitrogen/carbon allocation), showed a significant decrease for concentrations above 400 mg PHE kg^−1^ soil conditions compared to low-spiked conditions (0–100 mg PHE kg^−1^ soil) (Table S1).

### 2.3. Phenanthrene Dissipation

**Figure 2:**
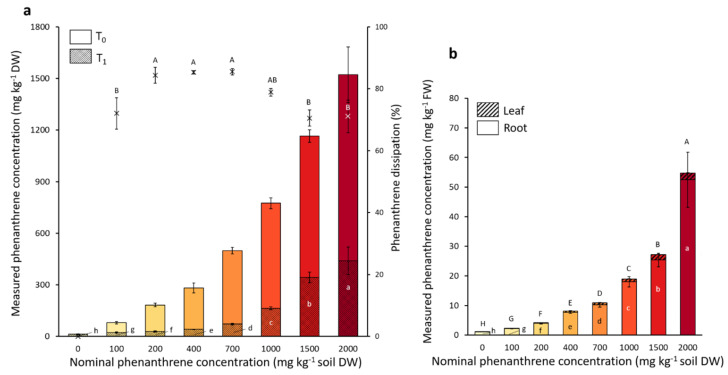
Measured phenanthrene concentrations in soil (**a**) and in poplar tissues (**b**). Each plot represents the mean data of four plant replicates cultivated at a different phenanthrene concentration. The T0 values were measured right after spiking the soil and before the addition of the plant to the pot. The T1 values were measured after four weeks of plant growth. The bars represent the standard deviation to the mean. Different letters indicate significant differences (*p* < 0.05) between the nominal phenanthrene concentrations after Kruskal-Wallis and Dunn *post-hoc* tests.

The microcosms were spiked with eight different nominal PHE concentrations ranging from 0 to 2000 mg PHE kg^−1^ soil. The initial PHE concentrations (T0) before the plant installation and also the final PHE concentrations after the four weeks of plant growth (T1) were measured, and the percentage of PHE dissipated calculated (Figure 2). Although 70.5 to 91.5% of the nominal PHE concentration was recovered at T0, the PHE gradient was well represented. The majority of the initial (T0) spiked PHE contamination dissipated within four weeks (Figure 2), and the PHE dissipation percentage ranged from 70% to 85% depending on the PHE condition. 

The dissipation percentage of three intermediate PHE conditions (200–400–700 mg PHE kg^−1^) were significantly higher (84.3%, 85.3% and 85.5%, respectively) than the two highest (1500 and 2000 mg PHE kg^−1^) concentrations with a dissipation percentage of 70.5% and 71.1%, respectively. The remaining PHE concentration at T1 was significantly different in all PHE conditions still representing a gradient. The remaining PHE was also tested for its bioavailability (data not shown). The bioavailable fraction represented 12.1%, 42.8% and 51.9% of the PHE in the conditions spiked with 1000, 1500 and 2000 mg PHE kg^−1^, respectively, while it represented only 2.0 to 2.6% of the remaining PHE in the low-spiked conditions (0–200 mg PHE kg^−1^). The PHE concentration recovered in the roots and leaves was also measured. PHE concentration in the plant was proportional to that of the soil. For the two highest PHE soil concentrations (1500 and 2000 mg PHE kg^−1^), we found up to 25.4 and 52.5 mg PHE kg^−1^ plant FW in the roots and 1.9 and 2.3 mg PHE kg^−1^ plant FW in the leaves, respectively. At these two concentrations it represented 2.2% and 3.4% of the initial measured PHE concentration present in the soil transferred to the roots, respectively, whereas for the leaves it represented only 0.16% and 0.15% of the initial measured spiked concentration, respectively. For every PHE condition there was between 15 to 20 times more PHE found in the roots compared to the leaves.

### 2.4. Fungal Community Diversity

A total of 4,298,485 ITS reads were obtained from 128 samples (32 samples for each compartment: soil, roots, stems, and leaves). After quality filtering and plant reads discarding, a total of 633,043, 669,654, 406,252 and 22,205 fungal ITS sequences from soil, roots, stems and leaves were kept for analysis, respectively. To compare the fungal richness and diversity among samples, data were rarefied to 22,188, 23,245, 3931 and 138 reads per sample for soil, roots, stems and leaves, respectively. Rarefaction curves indicated that the sequencing depths were sufficient to reliably describe the fungal microbiome for each compartment (Figure S2). A total of 3657 OTUs were observed for the four compartments, with 73 OTUs (2%) shared between all plant compartments (Figure S3). OTUs that were unique to each compartment represented 84, 32, 18 and 10% of the total OTUs for the soil, root, stem and leaf compartments, respectively (Figure S3).

**Figure 3:**
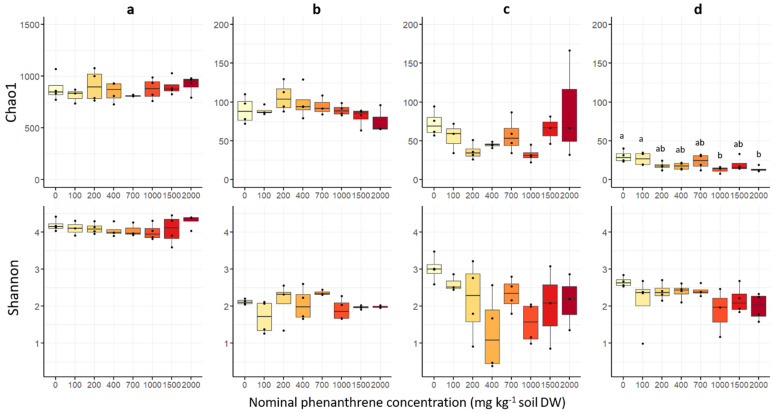
Fungal alpha diversity indices of soil, root, stem and leaf samples of poplar grown along a phenanthrene contamination gradient. (**a**) Soil (**b**) roots (**c**) stems and (**d**) leaves data. The indices (Shannon diversity and Chao1 richness) were calculated using the Phyloseq package on R. The data represents the distribution of measured parameters from three to four plant replicates per condition. Different letters indicate significant differences (*p* < 0.05) after Dunn’s *post-hoc* test.

The estimated fungal richness (Chao1 index) was different among plant compartments (Figure 3), and was significantly the highest in soil, then in roots, stems and finally in leaves (Table S2). Fungal richness was not significantly affected by the soil PHE contamination, except in the leaf compartment where a significant decrease in the Chao1 index was observed between low (0–100 mg PHE kg^−1^) and high (1000 and 2000 mg PHE kg^−1^) PHE conditions. The Shannon diversity indices (Figure 3) were significantly higher in soil samples than for the plant samples, and no significant difference among the three plant compartments was observed (Table S2). Similarly, for the other alpha diversity indices (InvSimpson, Simpson, ACE, Fisher), no significant difference was observed along the PHE gradient (Table S3).

**Figure 4:**
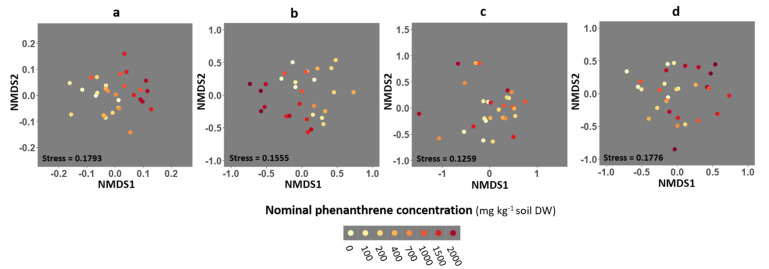
Non-metric multidimensional scaling (NMDS) ordination of fungal community composition associated with the rhizosphere soil, and the roots, stems and leaves of poplar grown along a phenanthrene contamination gradient. (**a**) Soil (**b**) roots (**c**) stems and (**d**) leaves data. Plots were drawn using the Bray-Curtis dissimilarity measure over 1000 iterations. Each point represents the fungal community of a given sample.

Three-dimensional non-metric multidimensional scaling (NMDS) analysis from the Bray-Curtis dissimilarity matrix was performed to compare the fungal community composition of the four different compartments (soil, roots, stems and leaves) as a function of the PHE concentration (Figure 4 and Figure S4). PERMANOVA were performed to test if the fungal community structures differed along PHE conditions (groups). A significant effect of the PHE concentration on community diversity was found in soil (PERMANOVA: *p* = 0.001, *R*^2^ = 0.364) and root (PERMANOVA: *p* = 0.038, *R*^2^ = 0.337) compartments. The PHE concentration explained 36 and 34% of the variance in the fungal communities from the soil and roots, respectively. No significant effect of the PHE concentration was observed on the community composition from the stem and leaf compartments (PERMANOVA: *p* = 0.67, *R*^2^ = 0.234; *p =* 0.055, *R*^2^ = 0.273, respectively).

**Figure 5:**
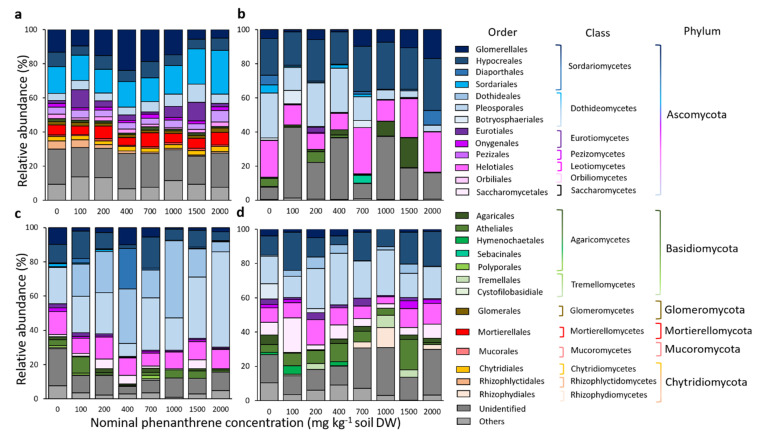
Taxonomic composition of the fungal communities of rhizosphere samples from soil (**a**), roots (**b**), stems (**c**) and leaves (**d**) of poplar, grown along a phenanthrene contamination gradient. The top 15 most abundant orders are represented, and the remaining diversity was categorized as ‘others’ due to their minor (<5% for soil samples or <1% for plant samples) relative abundances or ‘unidentified’ for non-affiliated taxa. Values are means of three to four replicate samples.

Fungal community diversity was explored at the phylum, class, order and genera levels. When considering the top 15 most abundant orders for each compartment, they belonged to six different phyla, 15 classes and 26 orders (Figure 5). The remaining diversity and non-affiliated taxa were categorized as ‘others’ and ‘unidentified’, respectively. In every compartment, the RA of *Basidiomycota* and *Ascomycota* were negatively correlated to each other (Figure S5, Soil: Pearson correlation r = −0.47; Roots: r = −0.73; Stems: r = −0.84; Leaves: r = −0.71). Sordariales and Glomerellales (RA of 17 and 13.2%, respectively), belonging to the Sordariomycetes and Ascomycota, dominated the soil samples. Their abundances were respectively, positively and negatively correlated to the PHE gradient (Pearson correlation r = 0.76 and −0.43; Figure S5). The relative proportion of Mortierellales and Chytridiales, two orders only detected in soil samples, was not impacted by the PHE gradient, while Rhizophlyctidales belonging to the Chytridiomycota (2.1% of the RA) were negatively correlated to the PHE concentration. Seven different genera were also positively correlated to the PHE gradient in the soil, namely: *Absidia, Acremonium, Alternaria, Aspergillus, Cadophora, Penicillium* and *Trichoderma* (Figure S5). For the root compartment, the four most abundant orders belonging to the Ascomycota were Hypocreales, Helotiales, Pleosporales and Glomerellales (RA of 24.2, 17.3, 14.8, and 7.3%, respectively). The relative proportion of the first two was not correlated to the PHE gradient, while the RA of Pleosporales was negatively correlated to the PHE concentration (Pearson correlation r = −0.49). Conversely, the RA of Glomerellales was positively correlated to the PHE concentration (Pearson correlation r = 0.65) including *Colletotrichum* (Pearson correlation r = 0.63). In the stem and leaf compartments, Pleosporales was the most abundant order (RA of 26.8 and 20.3%, respectively) and in stems it was positively correlated to the PHE concentration (Pearson correlation r = 0.43), contrary to the root compartment. A higher proportion of Basidiomycota was detected in leaves compared to other compartments. The proportion of unclassified fungi was positively correlated to the PHE concentration (Pearson correlation r = 0.37).

Fungal communities were explored concerning their trophic mode and how it was affected throughout the PHE contamination gradient. A trophic mode could be attributed to 14.9% to 52.6% of the fungal diversity depending on the condition (compartment, PHE concentration). No significant difference could be observed for any trophic mode across the four compartments studied and the PHE gradient (Figure S6).

### 2.5. Indicator Species Linked to the PHE Contamination

**Figure 6:**
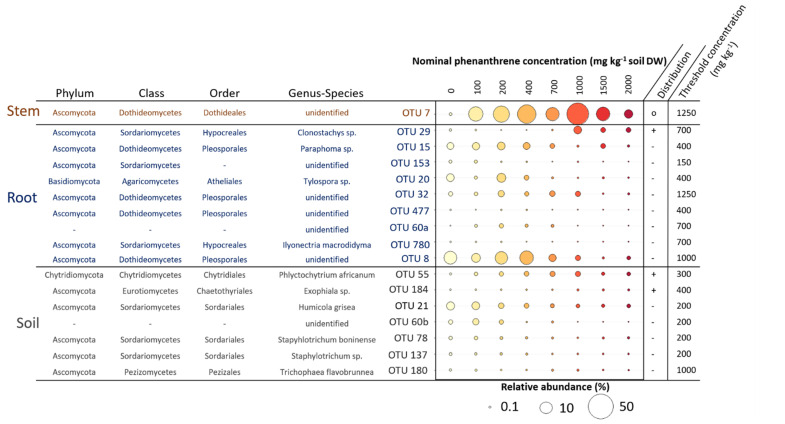
Mean relative abundance (RA) of different operational taxonomic units (OTUs) identified as indicator species (IS) in soil, root and stem samples of poplar whose relative abundance increased or decreased along the phenanthrene contamination gradient. Taxonomy at phylum, class, order, genus and species level of these indicator species is shown. A TITAN2 package in R was used to identify indicator species and species-specific response to the phenanthrene gradient. Only the species with purity > 0.9 and reliability > 0.9 were considered as credible indicator species for the gradient and thus kept. The distribution of the indicator species along the gradient is represented on the side of the plot (−: decreasing; +: increasing; o: wedge-shaped distribution). The threshold phenanthrene concentration value represents the specific change point where the indicator species is expected to change abruptly (change-point).

We searched for indicator species (IS) having their relative abundance increasing or decreasing along the PHE gradient in the four compartments studied (Figure 6). We identified a total of 19 OTUs that were significantly impacted by the PHE gradient; seven were found for the soil, nine for the roots, one for the stems. In the soil, five OTUs had their RA decreasing when the PHE concentration increased. The change point for their distribution was at 200 mg PHE kg^−1^ except for one decreasing from 1000 mg PHE kg^−1^. These OTUs belonged to the Sordariales (*Humicola grisea*, *Staphylotrichum boninense*, *Staphylotrichum* sp.), to unidentified fungi, and the last to Pezizales (*Trichophaea flavobrunnea*). The two last soil IS belonged to Chytridiales and Chaetothyriales, with RAs increasing along the gradient from 300–400 mg PHE kg^−1^. Eight of the IS for the root compartment had their RA decreasing along the PHE gradient at diverse change points from 150 to 1250 mg PHE kg^−1^ and half belonged to the Pleosporales, one to Hypocreales, and one to Atheliales. Only one IS for the roots belonging to Hypocreales (*Clonostachys* sp.) had its RA increasing with the PHE concentration (change point at 700 mg PHE kg^−1^). In the stems, one OTU was identified as IS; it belonged to the Dothideales and its RA, representing 0.3% at 0 mg PHE kg^−1^, increased up to 54% from 0 to 1000 mg PHE kg^−1^ and then decreased (change point at 1250 mg PHE kg^−1^) to 6% at 2000 mg PHE kg^−1^.

## 3. Discussion

We explored the impact of a PAH contamination gradient on poplar endophyte fungal diversity. Two hypotheses were made. First, the soil community diversity directly exposed to PAH will be modified and as soil microbial communities is the first reservoir of endophyte communities [29], the PAH contamination would lead to modifications of the plant tissue microbiomes [30]. Second, the response of plants to abiotic stress such as PAH contamination modify both plant physiology and metabolic signaling leading to the selection of different endophytic microbiome communities. We assumed that the level of soil contamination would affect plant growth parameters and fitness as well as fungal communities from soil, root, stem and leaf compartments in different ways throughout an increasing soil PHE contamination gradient. 

In our study, we observed a significant impact of the PHE contamination on the measured plant parameters after a certain concentration (above 400 mg kg^−1^), but it did not increase any further with the gradient. We deliberately limited the PHE concentration to 2000 mg kg^−1^ to prevent an excessive initial toxicity that would be lethal to the plants (preliminary data not shown). PAH contamination reduced root and leaf biomass, a finding already reported in several studies performed on various plant species in controlled conditions [22,31]. The transfer of PHE to the plant and its accumulation highlighted would result in local cell death and may explain the decrease in biomass at high PHE concentrations. Production of endogenous stress-related molecules such as reactive oxygen species (ROS) can also increase the cell damage locally [32]. The decrease in chlorophyll content could also be linked to the ROS production directly or indirectly. A decline in photosynthesis could be a defensive response to reduce ROS by-product synthesis in chloroplasts [33]. Studies investigating plant response to a PAH contamination gradient usually observed a linear response of the parameters studied (biomass, enzymatic activities, fitness parameters) to the contamination. However, unlike here, these studies were done with a different experimental set-up on *Arabidopsis* and wheat seedlings in controlled and sterile environments [22,34]. Our results suggest that at some point the plant can cope with the abiotic stress even if its growth and development was still impaired [35]. At the end of the growth period, there was still a significant amount of bioavailable PHE in the highly contaminated conditions compared to the low-spiked conditions which could explain the increased effects observed at higher concentrations. 

We observed shifts of the fungal community composition in the soil and the roots due to the PHE gradient as shown in the literature in the case of petroleum contamination on the willow rhizosphere [36]. However, in our study there was no reduction in diversity and richness indices level for the fungal communities of the belowground habitats contrary to what has been reported in previous studies with petroleum hydrocarbon contamination [36,37,38]. The plant produces a wide range of molecules providing microorganisms with a favorable physical and chemical environment (increased microbial biomass and activity, higher nutrient concentrations). This will limit the impact of the contamination on the microbial communities [30,36]. PHE contamination therefore causes the replacement of sensitive species by tolerant ones without affecting the richness and level of diversity. Moreover, these community composition shifts did not seem to impact the functional diversity as shown by the lack of difference in the fungal trophic modes.

The overall abundance of Sordariales in the soil was positively correlated to the contamination gradient (even though the abundance of some IS decreased). This order was already described in a PAH-contaminated soil and its abundance was also positively correlated to the contamination level [39]. Moreover, in the soil, *Absidia, Acremonium, Alternaria, Penicillium, Trichoderma, Aspergillus* and *Cadophora,* that are mainly described as saprotrophic, plant pathogenic or plant beneficial genera, were all positively correlated to the contamination gradient. All these genera have already been described as potential hydrocarbon biodegraders in contaminated soils or as strains improving plant growth and tolerance to abiotic stresses [40,41,42,43,44,45]. We also identified a few indicator species increasing along the PHE gradient with some interesting ones (OTU29: *Clonostachys* sp.; OTU55: *Phlyctochytrium africanum*; OTU184: *Exophiala* sp.) as they were already described as potential hydrocarbon degraders [46,47,48].

Glomerellales and its associated genus *Colletotrichum,* composed of mainly opportunistic phytopathogens species [49], were positively correlated to the PAH contamination in the roots. The impact of the contamination on the plant physiology and the soil microbial community could lead to a shift of the endophyte communities towards opportunistic phytopathogens communities. The physiological response of plants to PHE contamination might lead to a suitable environment for phytopathogen species and thus leading to a colonization of opportunistic species from the soil [29].

We observed a majority of indicator species belonging to the soil and root compartments, confirming that the impact of the PHE contamination was mainly found in the belowground habitats. Moreover, we observed that most of the indicator species had their relative abundance decreasing when the contamination increased. This finding could indicate that the contamination had a toxic effect on fungal communities at the OTU level. In the root compartment, several IS belonging to the Pleosporales for which some species were evidenced as PGP fungi like *Paraphoma* sp. were identified in our study [50]. If PHE contamination affects the colonization of PGP fungi capable of protecting plants, it could partly explain the adverse effect of the contaminant on plant growth and physiology.

We also investigated the fungal communities in the aboveground compartments (stems and leaves). Indeed, this was reported in the present study for poplar and previously in 12 other plant species [7]. PHE can be transferred to aboveground tissues. The fungal community composition was not affected, contrary to that of the belowground habitats. This result suggests that the toxic effect of PHE on the fungal communities could be limited to the roots and the soil as the PHE concentration detected in leaves was limited (up to 20 times less in the leaves compared to the roots). Even though plant fitness and biomass were affected on the aboveground parts at high PHE concentrations, fungal communities were weakly impacted. The plant would represent a physical and chemical barrier for the endophyte communities and protect them against contamination [12], and its stress response is not strong enough to induce variations in fungal communities.

We also observed a limited impact of the PHE contamination on the fungal richness and diversity indices in the aboveground parts except for the fungal richness in leaves, which decreased along the PHE gradient. Previous studies have shown that endophytic fungi of the phyllosphere can play an important role in enhancing plant health, acting as biocontrol agents against pathogens [51]. The plant’s internal environment can be modified by PHE contamination [22]. These changes of the host physiology and metabolome can cause shifts in microbial colonization and communities’ assembly [52]. Thus, a decrease in the fungal richness combined with the impact of the PHE contamination might have caused an even greater weakening of the plants in the aboveground habitats. The Pleosporales were positively correlated with the presence of the contamination in stems, whereas *Oculimacula* was negatively correlated in both stems and leaves. Pleosporales have often been reported in environments with high hydrocarbon contamination [12,53], while *Oculimacula* species, belonging to dark septate endophytes, have been isolated from extreme environments (low temperature, permafrost, long periods of darkness and light [54].

A single indicator species (OTU7), belonging to Dothideales, was significantly correlated to the PHE gradient in the stems and was the major fungal taxa in several PHE conditions (between 100 and 2000 mg PHE kg^−1^). Species belonging to Dothideales have not usually been found in hydrocarbon contaminated sites, even if they were found in environments under other abiotic stresses like high salinity or extreme temperatures. These heavily melanized fungi are usually highly resistant to abiotic stresses [55,56]. This could indicate that the conditions in the stems are quite harsh (due to PHE transport or plant physiology modification) and only highly tolerant fungi could thrive in this environment up to a certain point. The low amount of indicator species in the aboveground part confirmed that even at the OTU level, the impact of the PHE contamination is very limited. It seems that even though a transfer of PHE occurred in the aerial tissues and even if plant growth is affected from 400 mg kg^−1^ of PHE, its impact is very limited on the fungal communities in the aboveground habitats.

## 4. Material and Methods

### 4.1. Soil Sampling and Characteristics

The soil was sampled at 2–20 cm depth in a natural attenuation area of a brownfield site that formerly hosted a coking plant during the 20th century (49°12.54′ N, 5°59.528′ E, Homécourt, Grand Est region, France). Soil was air-dried and sieved to 2.4 mm and stored at room temperature until experimental set up. Physico-chemical analyses were performed at the Laboratoire d’Analyses des Sols (INRAE, Arras, France). It is a sandy soil (72.1% sand, 21.5% silt and 6.4% clay), with a pH_water_ of 8.2 and total organic carbon and total nitrogen concentrations of 108 and 2.55 g kg^−1^ DW soil, respectively. The sum of the 16 US-EPA priority PAHs was 233 ± 45 mg kg^−1^ DW soil and phenanthrene (PHE) concentration was 11.7 ± 1.6 mg kg^−1^ DW.

### 4.2. Soil Spiking with Phenanthrene

To set up microcosms, the soil was mixed with 10% (*w*/*w*) sand to increase its porosity and spiked with increasing phenanthrene (PHE) concentrations. The soil was spiked with PHE to reach final nominal concentrations of 0, 100, 200, 400, 700, 1000, 1500 and 2000 mg kg^−1^ DW soil. Aliquots of 100 g DW soil were prepared and a PHE-hexane solution was added to 1:10 of the soil aliquots for spiking (for 0 PHE condition soil was spiked with hexane without PHE). Hexane was evaporated under a fume hood for 48 h and then the spiked soil was homogenized with the remaining 9:10 of the soil aliquot. The mixed spiked soil was used for microcosm experiments.

### 4.3. Plant Pre-Culture and Microcosm Experiments

The plant cuttings of *Populus* × *canadensis* (hybrid of *P. nigra* × *P. deltoids;* cuttings provided by professional plant producer: SARL France Peupliers; poplar hybrid originally coming from public research centre of Casale Monferrato, Italy), with equal size (1 cm diameter and 10 cm height), were grown hydroponically in perlite and tap water for four weeks until rooting (3 to 10 cm long roots) was observed. The cuttings were then transplanted into the soil of the microcosms. A first experiment was conducted to explore the fungal colonization of roots through microscopy. In this experiment, only five PHE concentrations were used to reach final nominal concentrations of 0, 200, 700, 1000 and 1500 mg kg^−1^ DW soil. Plants were grown in triplicate in pots containing three cuttings per pot. The second experiment consisted of microcosms conducted with the eight different PHE concentrations. Soil was added to 100 mL glass beakers and wrapped with aluminum foil. Four replicates per PHE concentration were used, representing a total of 32 microcosms. One poplar cutting per microcosm was planted, and soil was watered to 100% of its water retention capacity (WRC) for the first three days and then maintained at 70% WRC using tap water. The experiments were conducted for four weeks in a growth chamber under controlled conditions (22 °C/18 °C with 16 h/8 h day/night, 250 µmol photons m^−2^ s^−1^, 80% relative humidity). After four weeks of growth, before plant harvest, the flavanol, anthocyanin and chlorophyll contents were measured using a Dualex FORCE-A (Orsay, France) on the second row of leaves.

### 4.4. Fungal Colonization and Microscopy

Fungal root colonization was assessed through two different dyes (Trypan blue and WGA) on poplar roots from the first experiment. After microcosm harvesting, the root samples were washed three times with distilled water and stored in 70% ethanol. Apex (<1 mm diameter) and aged roots (1–2 mm diameter) were incubated at 95 °C in 10% (m/v) KOH for 20 min. After three washes in distilled water, root systems were incubated for 4 h with 1% HCl. The HCl was removed after three washes with distilled water. The root system was soaked in Trypan Blue (0.1%) and incubated for 20 min at 90 °C. After three washes with distilled water, the roots were incubated overnight in 20% acidified glycerol (1% HCl). After solution removal, the roots were then stained with a second dye using 50 µg/mL WGA-AF^®^488 (Wheat Germ Agglutinin, Invitrogen, France) solution in 1X PBS and incubated at room temperature for 30 min. Finally, the roots were washed in distilled water and mounted between slides and cover slips with a drop of 20% glycerol for observation under an optical/epifluorescence microscope (Nikon Eclipse 80i). Light microscopy was used to investigate for Trypan blue-stained mycorrhizal and melanized fungi and fluorescent observation was done to visualize fungal cell walls stained with WGA (λ_excitation_: 405 nm; λ_emission_: 500–550 nm). The fungal colonization percentage was calculated using Trouvelot’s method [57] on an average of 20 root sections (1 cm long) per plant replicate.

### 4.5. Harvest of Soil, Root, Stem and Leaf Samples from the Second Experiment

After four weeks of growth, the number of leaves and the size of the plants of each microcosm were measured. The plants were removed from the pots and the rhizospheric soil was recovered by slightly shaking the roots. Rhizospheric soil was stored at −80 °C in glassware. Roots, stems and leaves were collected separately, the remaining soil adhering to the roots was gently removed with a fine paintbrush and plant materials were surface sterilized (70% ethanol for 1 min, 4% sodium hypochlorite for 1 min, 70% ethanol for 30 sec and finally washed in sterile distilled water three times). Plant material was gently soaked with paper towels, weighted and directly flash-frozen and kept at −80 °C until DNA extraction and PHE analysis. Root, stem and leaf samples were crushed in liquid nitrogen for homogenization before use for further analyses.

### 4.6. Phenanthrene Analysis

For each PHE condition of the second experiment, three replicates were directly stored at −80 °C to analyze T_0_ phenanthrene spiking right after mixed spiked soil preparation and served as controls of the measured initial PHE concentration. Moreover, rhizosphere soil samples (T1 after 4 weeks) were also analyzed. All samples were freeze-dried and ground. PHE was extracted from 2 g DW soil using dichloromethane (DCM) and a high-pressure (100 bars) and high-temperature (130 °C) DIONEX^®^ 200 ASE (Accelerated Solvent Extractor) automated extractor. DCM was evaporated from the PAH extracts under nitrogen flow and replaced by acetonitrile for PAH analysis. PAH extracts were analyzed using a reverse-phase chromatography UHPLC Ultimate 3000 RSLC system (Dionex) equipped with a Diode Array Detector and a Zorbax Eclipse PAH RRHD column (100 mm × 2.1 mm, 1.8 mm-Agilent). The mobile phase was a mixture of water/acetonitrile in gradient mode, with a flow rate of 0.42 mL min^−1^. The wavelength used for detection was 254 nm.

The bioavailable soil PHE concentration was measured from 1 g of soil (fresh weight) with 50 mM hydroxypropyl-β-cyclodextrin (ThermoFisher Scientific, Illkirch, France). After two liquid:liquid extractions with dichloromethane, the extracts were pooled and evaporated under nitrogen flux and resuspended in acetonitrile. The extracts were filtered at 0.45 µm (PTFE, VWR) and analysed by UHPLC as described above.

PHE concentration was also measured in plant tissues. Two hundred mg of frozen powder of the roots and leaves were extracted with 1 mL of methanol at −20 °C over 16 h. After centrifugation (2 °C, 13,000 rpm), supernatants were dried in a speed vacuum system. A second extraction with 1 mL of ethyl acetate was performed and the supernatant was added to the first pellet, dried and resuspended in 300 µL of methanol. Samples (1 µL) were analyzed by GC-MS (436-GC, Bruker; column 30-m, 0.25-mm, 0.25 µm Rxi-5Sil MS, Restek and retention gaps Rxi 5-m, 0.25-mm, Restek) with He carrier gas inlet pressure programmed for constant flow of 1 mL/min and mass spectrometric detector (SCION TQ, Bruker; 70 eV). The injector temperature was set at 310 °C with a pressure pulse (30 psi, 0.7 min). GC was carried out with temperature programmed injection at 70 °C, oven 0.7 min at 70 °C, raised by 30 °C/min to 180 °C and then 10 °C/min to 300 °C and held for 9 min at 300 °C. Absolute quantification was achieved by comparison of sample signals with a dose–response curve established with pure phenanthrene. Compound specific detection was performed in Multiple Reaction Monitoring mode using transitions 178 > 152 and 178 > 176, with collision energy 30 V. The data analysis was performed with the Bruker MS Data Review software (ver 8.2.1).

### 4.7. DNA Extraction and ITS Sequencing

DNA was extracted from approximatively 50 mg of powder of roots, stems and leaves using the DNeasy Plant Mini Kit (Qiagen, Hilden, Germany). DNA from approximatively 300 mg fresh weight of rhizosphere soil was extracted using the FastDNA Spin Kit for soil (MP Biomedicals Europe, Illkirch, France). DNA concentration and quality were estimated using a NanoDrop™ One spectrophotometer (ThermoFisher Scientific, Illkirch, France). A two-step PCR approach was used to barcode-tag templates. For the first PCR, ITS3 and ITS4 primers (0.2 µM) targeting the fungal ITS2 rRNA region and merged to specific tags for the second PCR, were used [58]. To inhibit plant material amplification, we added peptide nucleotide acid (PNA) blocker oligos (Pangene, Korea) targeting plant 5.8S nuclear rRNA [59]. The PNA sequence was designed specifically for *Populus* species [60]. A high-fidelity Phusion polymerase (ThermoFisher Scientific, Illkirch, France) was used and the thermal cycler conditions for the first PCR were 98 °C for 3 min followed by 35 cycles of 98 °C for 10 s, PNA annealing at 78 °C for 10 s, primers annealing at 54 °C for 30 s, 72 °C for 30 s and a final extension step at 72 °C for 5 min. PCR products were checked using agarose gel electrophoresis (1% in TAE). Amplicons from the first PCR were sent to Microsynth AG (Balgach, Switzerland) for the second PCR including barcodes, the sample library preparation and 2 × 300 bp paired-end MiSeq sequencing (v.3, Illumina). Reads have been deposited in the SRA database under BioProject accession number PRJNA820614.

### 4.8. ITS Sequencing Data Analysis

Reads were processed using FROGS (Bioinfo Genotoul) [61]. The Fastq sequences were merged using Vsearch. Reads were filtered to discard sequences smaller than 250 bp. The remaining reads were clustered into operational taxonomic units (OTUs) based on the iterative Swarm algorithm (d = 1), then chimeras, singletons (OTUs containing only one sequence among all samples) and OTUs present in a single sample were removed. Sequences belonging to plant material were filtered using the ITSx function of FROGS. Per-sample rarefaction curves were produced to assess sampling completeness, using function *rarecurve*() in the package Vegan v2.5-7 in R (version 4.1.0) [62].Taxonomic assignment was performed with the UniteFungi8.2 database. Based on these, subsequent analyses of diversity and community structure were performed on data sets where samples had been rarefied with the Phyloseq package [63] to achieve equal read numbers according to the minimum number of total reads in any sample (22,188 reads for soil, 23,245 reads for roots, 3931 reads for stems and 138 reads for leaves). Samples with an insufficient number of sequences after rarefaction were removed (two samples for soil at 100 and 400 mg PHE kg^−1^ DW soil, two for roots at 700 and 2000 mg PHE kg^−1^ DW soil, three for stems at 100, 1500 and 2000 mg PHE kg^−1^ DW soil, and one for leaves at 1000 mg PHE kg^−1^ DW soil). The Alpha diversity indices (Chao1, Shannon, ACE, Simpson, Fisher) were calculated using the Phyloseq package, which was also used for fungal community composition and structure analysis. Each fungal OTU was further assigned to trophic groups of fungi using the FungalTraits database [64]. For each assignment, three confidence rankings (i.e., “possible”: suspected, “probable”: fairly certain, “highly probable”: absolutely certain) referring to previously peer reviewed data were given. Only the functional and morphological assignations with at least a “probable” confidence ranking were considered.

### 4.9. Statistical Analyses

Statistical analyses and data representations were performed using R software (R studio, v1.4.1106). Plant growth parameters, PHE concentration and fungal colonization data were compared by using a Kruskal-Wallis test (*p* < 0.05) followed by Dunn’s *post-hoc* test or one-way analysis of variance (ANOVA) followed by Tukey’s *post-hoc* test when non-parametric or parametric distribution (after Shapiro-Wilk test) were observed, respectively.

Fungal community composition (at OTU level) was compared among samples using nonmetric multidimensional scaling analysis (NMDS) based on Bray-Curtis dissimilarity matrices, analysis of similarity (ANOSIM) and permutational multivariate analysis of variance (PERMANOVA) using the pairwise Adonis package [65].

Average relative abundances (RAs) of taxonomic groups were achieved by averaging three to four replicates. These mean RA values were used to estimate differential abundance of taxonomic groups depending on PHE concentration using Kruskal-Wallis followed by the Benjamini-Hochberg false discovery rate (FDR) correction (non-parametric distribution of the data was checked with a Shapiro-Wilk test). Correlation matrices were made using the *corrplot* package on R [66] and using a *p* < 0.05 for significant correlation. A Venn diagram was made using the *Jvenn* plug-in from the INRAE Toulouse [67]. RAs of different indicator species from soil, root, stem and leaf samples were analysed using the TITAN2 [68] package in R. It was used to identify indicator species and species-specific response to the PHE gradient as well as threshold PHE concentration having an effect. Only the OTUs with purity > 0.9 and reliability > 0.9 were considered as credible indicator species and kept.

## 5. Conclusions

This study, thanks to an innovative experimental design (multiple PHE concentration and thee plant organs studied), has provided a singular view into poplar microbiome in a PAH-contaminated environment. We found out that the impact of the phenanthrene contamination on the fungal communities was different depending on the compartment studied. We observed a toxic effect of the contamination on the belowground habitats, (soil, roots) which resulted in a modification of the community structure, and sensitive species’ abundance was reduced while other species more tolerant and potentially involved in PHE degradation saw their abundance increase. The impact of the PAH contamination was negligible on the aboveground compartments. The endophytes present in the stems and leaves are probably less affected by the direct effect of PHE (limited concentration of PHE in leaves) but could be affected by a shift in the plant physiology due to the response to the contamination. This study highlighted a global overview of a complex fungal-plant system in the case of a soil PAH contamination for the first time. The findings could represent potential advancements for field applications using poplar associated fungi in PAH bioremediation systems. To have a better understanding, it would be interesting to go deeper in the physiological response of the plant to the contamination by investigating the oxidative stress response, as well as by using a multi-omics approach targeting the plant transcriptome, proteome and metabolome. A focus on other microorganisms like bacteria that potentially play a major role in a phytoremediation context could also be of great interest.

## Data Availability

Sequencing data are available in the SRA database under BioProject accession number PRJNA820614. The data presented in this study are available on request from the corresponding author.

## Supplementary Materials

The following supporting information can be downloaded at: https://www.mdpi.com/article/10.3390/ijms23115909/s1.
