# Supplementary material for: Response of Poplar and Associated Fungal Endophytic Communities to a PAH Contamination Gradient
Source: Int J Mol Sci. 2022 May 25;23(11):5909. doi: 10.3390/ijms23115909 (PMC9180295; doi:10.3390/ijms23115909)
Supplement: Supplementary file 1 [file ijms-23-05909-s001.zip › ijms-1701835-supplementary.pdf]

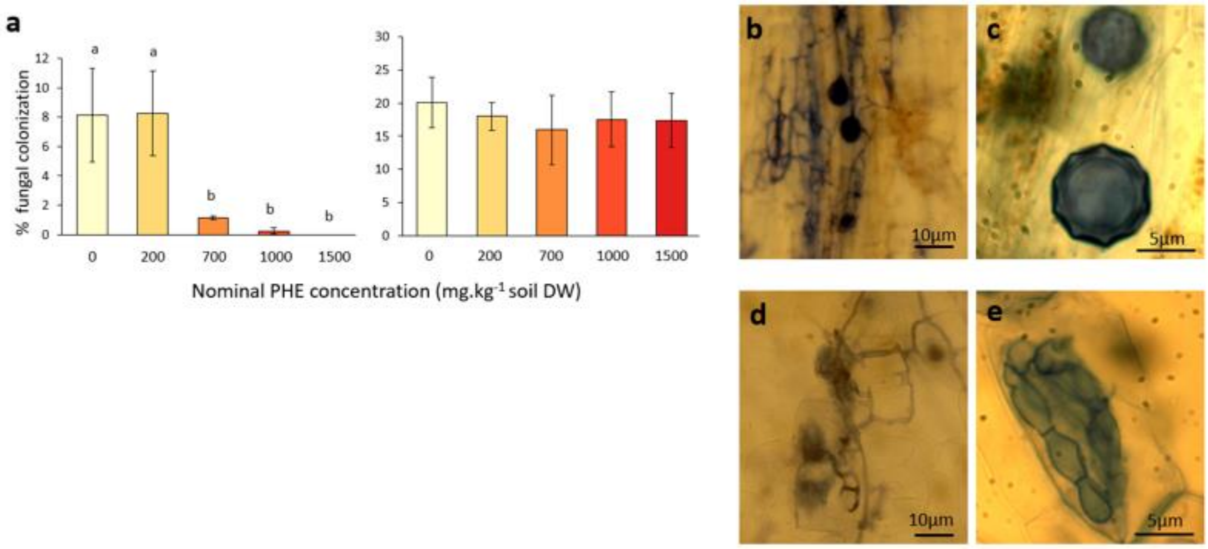

Figure S1. Observation of fungal colonization on *P. canadensis* roots cultivated in the presence of different phenanthrene (PHE) concentrations. (a) % of fungal colonization calculated according to Trouvelot's method. The data represent the mean of 20 root samples from 3 plant replicates per condition. The error bars represent the standard-deviation from the mean. Different letters indicate significant difference (P<0.05). Trypan blue-stained roots were observed by optical microscopy (b, c, d, e). A mix of vesicules. typical hyphae were observed in the 0 mg.kg^{-1} of soil PHE condition (b). Typical *Olpidium* structures (c) and arbuscules (d) were observed at low PHE concentratiosn (here 0 and 200 mg.kg^{-1} respectively). Sclerotium-like structures (e) were observed at high PHE concentration (here 1000 mg. kg^{-1}).

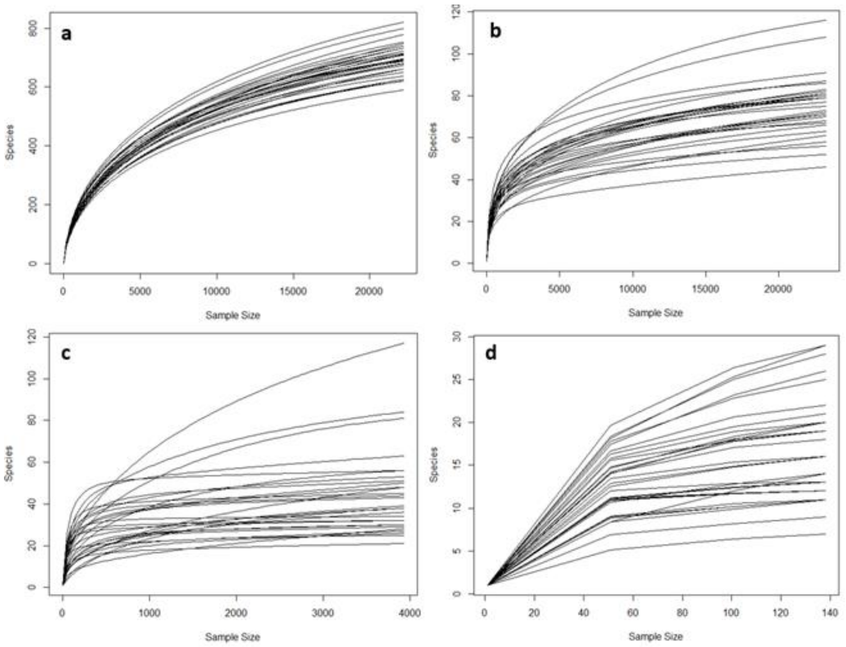

Figure S2. Rarefaction curves for the four compartments studied (soil, root, stem, leaf). The number of ITS reads from the soil, root, stem and leaf compartments were rarefied to 22,188, 23,245, 3,931 and 138 respectively. (a) Soil samples (b) Root samples (c) Stem samples (d) Leaf samples.

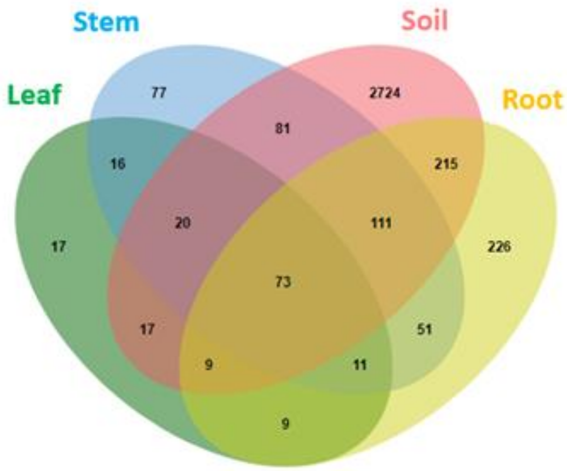

Figure S3. Four-way Venn diagram showing the OTUs specific or shared between the different plant compartments (soil, root, stem, leaf).

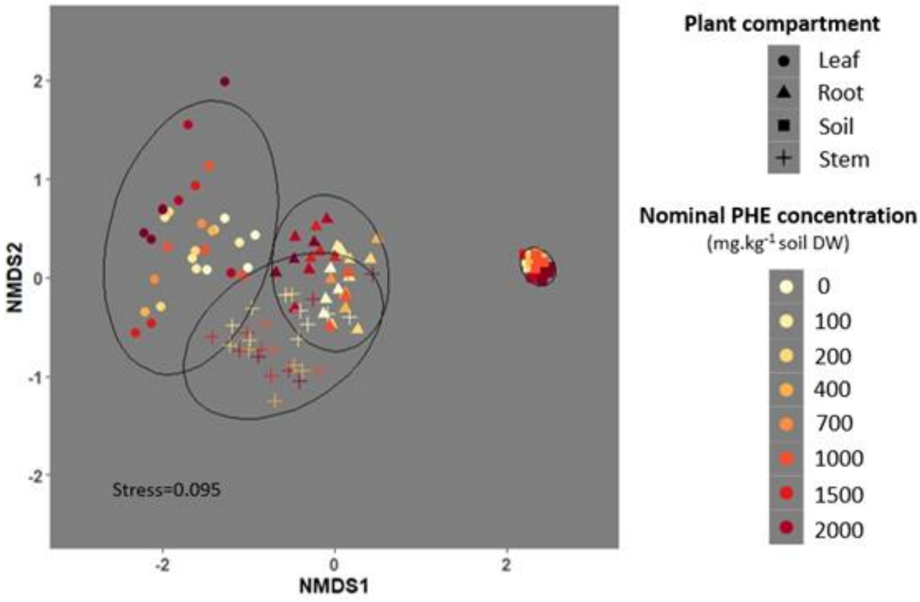

Figure S4. NMDS plot of fungal communities associated with the soil, roots, stems and leaves habitats of *P. canadensis* cultivated along a phenanthrene gradient. ANOSIM P = 0.001 and PERMANOVA P = 0.001. Pairwise comparison showed a distribution significantly (P <0.05) different between every compartment.

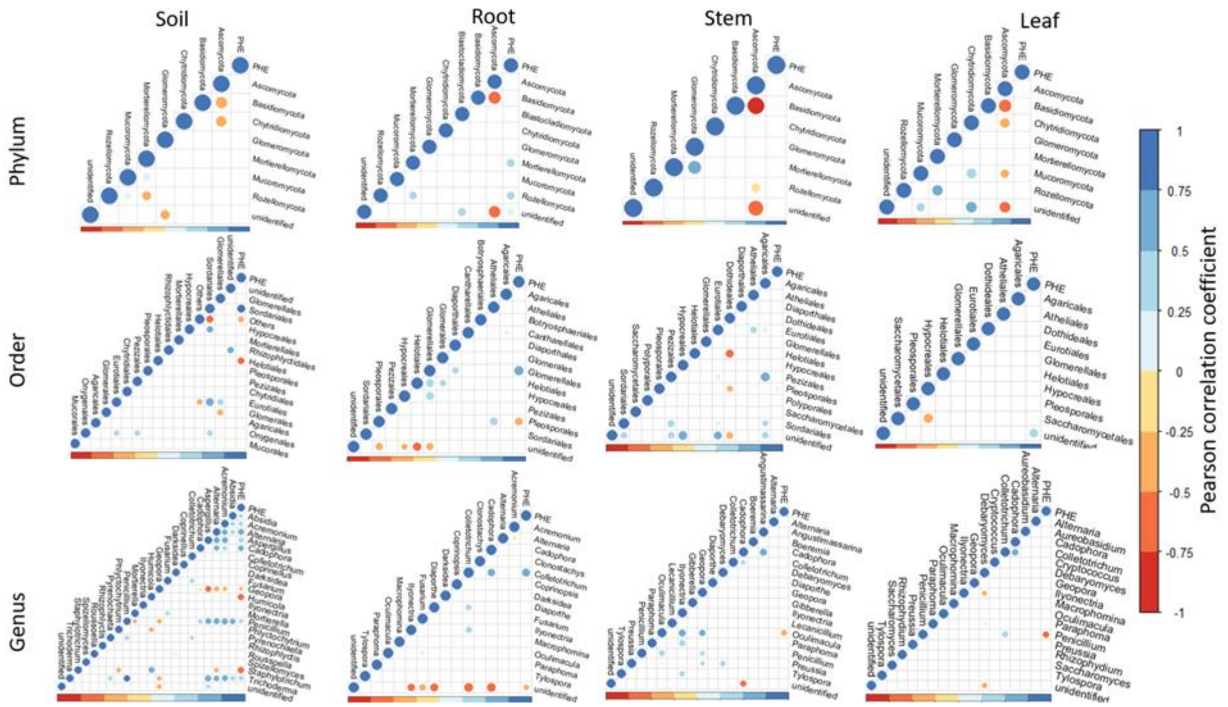

Figure S5. Pearson correlations matrixes for every compartment (soil, roots, stems, leaves) of fungal taxa (phylum, order, genus). A blue dot means that the Pearson correlation is positive, while a red dot means that the Pearson correlation is negative. The dots are shown for Pearson correlation with P <0.05.

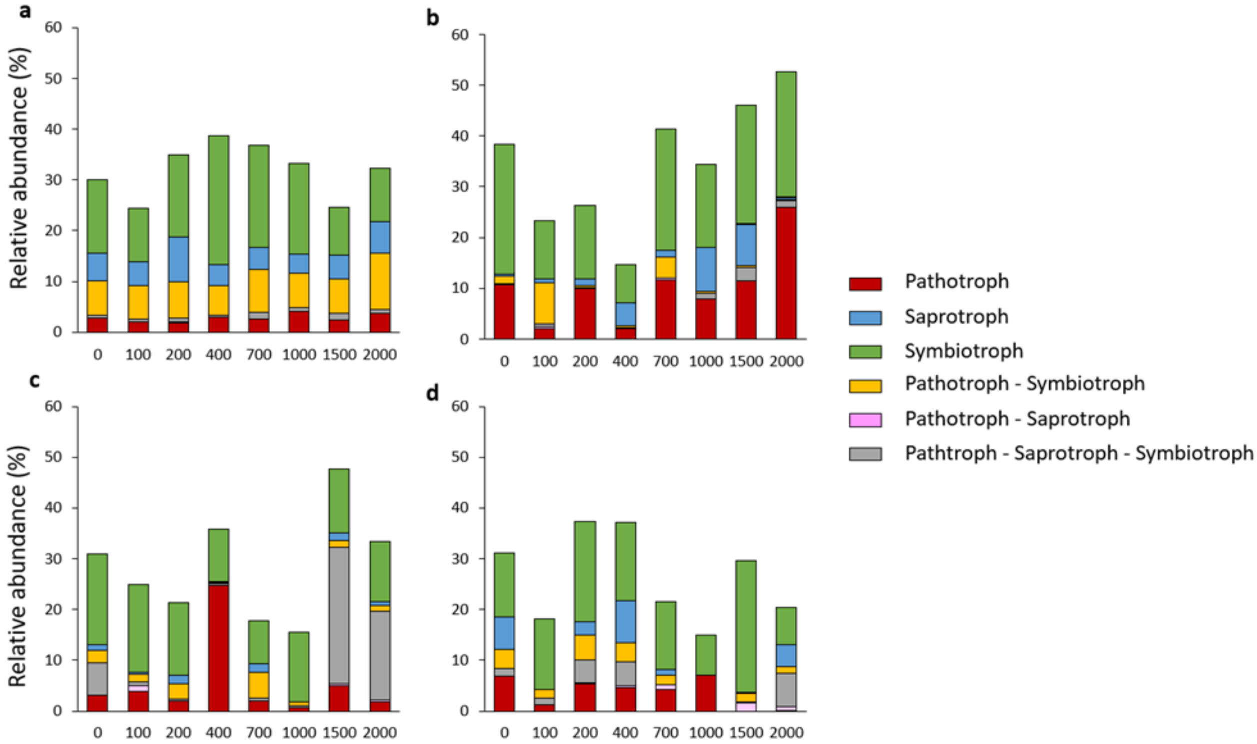

Figure S6. Relative abundance of the fungal trophic mode from the different compartments (soil, root, stem, leaf). (a) Soil (b) Root (c) Stem (d) Leaf. No significant difference was observed (Kruskal-Wallis, P <0.05). Trophic mode association was made using the FungalTraits database.

Table S1. Additional plant growth parameters measured before microcosm harvesting. Flavonols, anthocyanins and NBI content were measured using

DUALEX FORCE-A on the second row of leaves. Data are means of 3 to 4 plant replicates for each PHE concentration. Standard deviations to the mean are also represented. Any significant differences between the conditions are represented with different letters after a Kruskal-Wallis and Dunn *Post-hoc* test.

Nominal PHE concentration ($mg.kg^{-1}$)	Leaves number	Flavonols	Anthocyanins	NBI
0	12 ± 1.41 ^{a}	1.48 ± 0.18	0.25 ± 0.11	14.27 ± 6.75 ^{a}
100	12.25 ± 2.22 ^{a}	1.33 ± 0.04	0.19 ± 0.03	16.25 ± 3.27 ^{a}
200	10.75 ± 2.87 ^{ab}	1.4 ± 0.11	0.28 ± 0.03	10.87 ± 2.03 ^{ab}
400	9.75 ± 0.96 ^{ab}	1.46 ± 0.15	0.33 ± 0.05	8.3 ± 0.92 ^{bc}
700	8.25 ± 2.22 ^{b}	1.53 ± 0.19	0.29 ± 0.07	9.02 ± 2.68 ^{cd}
1000	10 ± 2.58 ^{ab}	1.59 ± 0.17	0.3 ± 0.09	8.37 ± 4.37 ^{cd}
1500	10.5 ± 2.38 ^{ab}	1.36 ± 0.23	0.29 ± 0.06	8.6 ± 2.11 ^{cd}
2000	9.5 ± 0.58 ^{ab}	1.5 ± 0.27	0.26 ± 0.02	9.43 ± 2.62 ^{d}

Table S2. Statistical differences of the estimated richness (Chao1) and diversity (Shannon) between the different plant compartments. Significant differences were identified following the use of Kruskal-Wallis and Dunny *post-hoc* tests.

a

	.y.	group1	group2	n1	n2	statistic	p	p.adj	p.adj.signif
1	Chao1	leaves	roots	31	30	6.229627	4.675468e-10	4.675468e-10	****
2	Chao1	leaves	soil	31	30	9.892857	4.470836e-23	4.470836e-23	****
3	Chao1	leaves	stems	31	29	3.326000	8.810204e-04	8.810204e-04	***
4	Chao1	roots	soil	30	30	3.633568	2.795293e-04	2.795293e-04	***
5	Chao1	roots	stems	30	29	-2.827068	4.697638e-03	4.697638e-03	**
6	Chao1	soil	stems	30	29	-6.429711	1.278470e-10	1.278470e-10	****

b

	.y.	group1	group2	n1	n2	statistic	p	p.adj	p.adj.signif
1	Shannon	leaves	roots	31	30	-1.8727538	6.110239e-02	6.110239e-02	ns
2	Shannon	leaves	soil	31	30	5.9510265	2.664659e-09	2.664659e-09	****
3	Shannon	leaves	stems	31	29	-0.4912844	6.232253e-01	6.232253e-01	ns
4	Shannon	roots	soil	30	30	7.7604289	8.464267e-15	8.464267e-15	****
5	Shannon	roots	stems	30	29	1.3544060	1.756069e-01	1.756069e-01	ns
6	Shannon	soil	stems	30	29	-6.3399754	2.298018e-10	2.298018e-10	****

Table S3. Additional alpha-diversity indexes calculated through R. Data represented are means of 3 to 4 plant replicates. Standard deviations to the mean are also represented and significative statistical differences are represented by different letters after Kruskal Wallis and Dunn *post-hoc* tests.

a

Soil	0	100	200	400	700	1000	1500	2000
Observed	695.8 ± 64.3	665.3 ± 40.1	688.8 ± 59.7	692.3 ± 49.4	689.3 ± 18.1	675.5 ± 65.5	704.3 ± 84.5	739.8 ± 47.0
Chao1	885.0 ± 128.7	813.6 ± 68.6	908.2 ± 155.0	862.3 ± 116.6	811.2 ± 8.0	914.7 ± 84.6	901.8 ± 87.2	919.0 ± 85.2
se.chao1	33.9 ± 8.9	28.2 ± 3.5	38.3 ± 13.5	30.9 ± 10.3	23.9 ± 3.2	36.7 ± 7.0	35.8 ± 2.2	31.6 ± 9.0
ACE	917.2 ± 141.3	847.6 ± 81.7	933.4 ± 155.4	892.4 ± 115.5	846.9 ± 20.7	891.7 ± 106.3	916.8 ± 110.0	957.4 ± 88.3
se.ACE	15.6 ± 1.8	14.9 ± 1.1	16.1 ± 2.1	15.4 ± 1.6	14.6 ± 0.5	15.5 ± 1.3	15.7 ± 1.1	16.0 ± 1.1
Shannon	4.2 ± 0.2	4.1 ± 0.2	4.1 ± 0.2	4.0 ± 0.0	4.1 ± 0.2	4.0 ± 0.2	4.1 ± 0.4	4.3 ± 0.2
Simpson	0.9 ± 0.0	0.9 ± 0.0	0.9 ± 0.0	0.9 ± 0.0	0.9 ± 0.0	0.9 ± 0.0	0.9 ± 0.0	1.0 ± 0.0
InvSimpson	21.5 ± 6.5	20.1 ± 4.1	20.0 ± 3.9	13.0 ± 1.4	17.7 ± 5.4	16.9 ± 3.6	18.1 ± 8.0	21.4 ± 3.9
Fisher	136.7 ± 15.7	129.2 ± 9.6	134.9 ± 14.5	135.8 ± 12.0	134.9 ± 4.4	131.8 ± 15.7	138.9 ± 20.9	147.4 ± 11.7

b

Root	0	100	200	400	700	1000	1500	2000
Observed	71.5 ± 6.9 abc	79.3 ± 0.5 ab	89.3 ± 15.2 a	88.5 ± 18.6 a	80 ± 7.9 a	72.5 ± 4.8 abc	62.5 ± 10.7 bc	54.3 ± 7.6 c
Chao1	93.4 ± 19.3	89.0 ± 5.6	112.3 ± 17.5	99.2 ± 21.1	94.8 ± 12.5	89.7 ± 7.1	76.3 ± 18.4	65.5 ± 17.8
se.chao1	11.2 ± 5.8	7.1 ± 4.0	10.2 ± 3.7	6.7 ± 1.9	9.4 ± 5.1	11.0 ± 4.7	13.4 ± 7.7	14.7 ± 6.6
ACE	92.5 ± 16.1 abc	90.3 ± 3.4 abc	109.8 ± 16.1 a	104.1 ± 21.5 a	100.6 ± 13.5 ab	90.6 ± 8.8 abc	78.2 ± 9.9 c	76.8 ± 14 bc
se.ACE	5.0 ± 0.6	4.7 ± 0.2	5.3 ± 0.4	5.1 ± 0.5	5.2 ± 0.7	4.5 ± 0.3	4.3 ± 0.5	4.1 ± 0.7
Shannon	2.1 ± 0.1	1.7 ± 0.5	2.1 ± 0.5	2.1 ± 0.4	2.4 ± 0.1	1.9 ± 0.3	2.0 ± 0.0	2.0 ± 0.0
Simpson	0.8 ± 0.0	0.6 ± 0.2	0.8 ± 0.1	0.8 ± 0.1	0.8 ± 0.0	0.7 ± 0.1	0.8 ± 0.0	0.8 ± 0.0
InvSimpson	5.2 ± 0.3	3.4 ± 1.7	6.1 ± 2.7	5.3 ± 2.9	6.3 ± 0.6	4.3 ± 1.5	4.7 ± 0.2	5.2 ± 0.5
Fisher	9.1 ± 1.0 abc	10.3 ± 0.1 ab	11.8 ± 2.3 a	11.7 ± 2.9 a	10.4 ± 1.2 a	9.3 ± 0.7 abc	7.8 ± 1.5 bc	6.7 ± 1.1 c

c

Stem	0	100	200	400	700	1000	1500	2000
Observed	60.8 ± 15.7	42.0 ± 13.1	33.0 ± 8.5	36.8 ± 7.6	52.3 ± 21.4	29.0 ± 6.2	44.7 ± 17.6	65.7 ± 21.4
Chao1	75.7 ± 26.2 a	46.9 ± 17.5 abc	36.3 ± 10.7 bc	44.8 ± 1.0 abc	73.0 ± 19.4 ab	27.0 ± 6.4 c	81.3 ± 17.8 ab	99.6 ± 19.4 ab
se.chao1	9.5 ± 7.1	11.7 ± 5.1	4.3 ± 3.6	7.7 ± 4.1	3.9 ± 3.1	3.8 ± 2.9	16.5 ± 13.1	10.9 ± 3.1
ACE	74.4 ± 19.0 a	61.7 ± 5.7 abc	35.5 ± 10.1 bd	43 ± 8.8 bcd	60.3 ± 23.2 abc	33.8 ± 8.8 d	61.5 ± 22.7 abcd	92.7 ± 23.2 ac
se.ACE	3.8 ± 0.8	2.7 ± 1.6	2.4 ± 0.3	3.0 ± 0.2	3.4 ± 1.1	2.7 ± 0.8	3.6 ± 0.7	4.7 ± 1.1
Shannon	3.0 ± 0.4	2.6 ± 0.2	2.2 ± 1.0	1.3 ± 1.0	2.3 ± 0.4	1.5 ± 0.5	2.0 ± 1.1	2.1 ± 0.4
Simpson	0.9 ± 0.0	0.9 ± 0.0	0.7 ± 0.3	0.5 ± 0.4	0.8 ± 0.1	0.6 ± 0.3	0.7 ± 0.3	0.7 ± 0.1
InvSimpson	12.8 ± 5.5	8.4 ± 2.5	8.2 ± 7.1	3.0 ± 2.4	5.8 ± 2.9	3.6 ± 2.3	6.0 ± 5.8	6.1 ± 2.9
Fisher	10.3 ± 3.2	6.6 ± 2.4	5.0 ± 1.5	5.6 ± 1.4	8.7 ± 4.3	4.3 ± 1.1	7.2 ± 3.3	11.7 ± 4.3

d

Leaf	0	100	200	400	700	1000	1500	2000
Observed	24.8 ± 5.1	19.5 ± 4.9	16.3 ± 4.0	16.5 ± 4.0	18.3 ± 5.6	11.3 ± 4.0	15.8 ± 8.2	12.0 ± 2.9
Chao1	30 ± 7.5 a	26.8 ± 8.5 a	17.9 ± 5.1 ab	17.4 ± 4.6 ab	23.4 ± 9.6 ab	12.7 ± 5.1 b	19.5 ± 9.2 ab	13.5 ± 3.7 b
se.chao1	5.1 ± 3.2	6.4 ± 7.7	2.3 ± 2.1	1.6 ± 1.2	5.6 ± 4.3	2.4 ± 2.1	4.6 ± 2.0	2.3 ± 2.3
ACE	30.7 ± 9.9	23.9 ± 7.2	18.5 ± 5.0	17.9 ± 5.4	21.5 ± 7.8	13.3 ± 5.0	21.4 ± 10.6	14 ± 3.8
se.ACE	2.7 ± 0.8	2.2 ± 0.5	2.0 ± 0.2	1.9 ± 0.2	2.1 ± 0.3	1.7 ± 0.2	2.3 ± 0.6	1.9 ± 0.3
Shannon	2.7 ± 0.1	2.1 ± 0.8	2.4 ± 0.2	2.4 ± 0.2	2.4 ± 0.2	1.9 ± 0.2	2.2 ± 0.4	2.0 ± 0.4
Simpson	0.9 ± 0.0	0.7 ± 0.3	0.9 ± 0.0	0.9 ± 0.0	0.9 ± 0.0	0.8 ± 0.0	0.8 ± 0.0	0.8 ± 0.1
InvSimpson	9.8 ± 2.8	6.4 ± 3.5	9.3 ± 2.4	8.6 ± 2.1	8.3 ± 0.3	6.1 ± 2.4	6.7 ± 1.8	6.0 ± 2.2
Fisher	8.9 ± 2.7	6.3 ± 2.3	4.9 ± 1.7	5.0 ± 1.7	5.8 ± 2.5	3.0 ± 1.7	4.9 ± 3.8	3.2 ± 1.1
